# Soluble (Pro)renin Receptor and Obstructive Sleep Apnea Syndrome: Oxidative Stress in Brain?

**DOI:** 10.3390/ijms18061313

**Published:** 2017-06-20

**Authors:** Kazuhiro Takahashi, Koji Ohba, Kazuki Tajima, Tsuguo Nishijima, Shigeru Sakurai

**Affiliations:** 1 Department of Endocrinology and Applied Medical Science, Tohoku University Graduate School of Medicine, Sendai, Miyagi 980-8575, Japan; k-ohba@med.tohoku.ac.jp (K.O.); tajikazu1991@yahoo.co.jp (K.T.); 2 Division of Behavioral Sleep Medicine, Iwate Medical University School of Medicine, Morioka, Iwate 020-0850, Japan; tsuguo@iwate-med.ac.jp (T.N.); ssakurai@iwate-med.ac.jp (S.S.)

**Keywords:** obstructive sleep apnea syndrome, oxidative stress, prorenin, (pro)renin receptor

## Abstract

(Pro)renin receptor ((P)RR) is a multi-functional molecule that is related to both the renin-angiotensin system (RAS) and vacuolar H^+^-ATPase (v-ATPase), an ATP-dependent multi-subunit proton pump. Soluble (P)RR (s(P)RR), which consists of the extracellular domain of (P)RR, is present in blood and urine. Elevated plasma s(P)RR concentrations are reported in patients with chronic kidney disease and pregnant women with hypertension or diabetes mellitus. In addition, we have shown that plasma s(P)RR concentrations are elevated in patients with obstructive sleep apnea syndrome (OSAS). Interestingly, the levels are elevated in parallel with the severity of OSAS, but are not related to the presence of hypertension or the status of the circulating RAS in OSAS. It is known that v-ATPase activity protects cells from endogenous oxidative stress, and loss of v-ATPase activity results in chronic oxidative stress. We hypothesize that hypoxia and subsequent oxidative stress, perhaps in the brain, may be one of the factors that elevate plasma s(P)RR levels in OSAS.

## 1. Introduction

**Figure 1:**
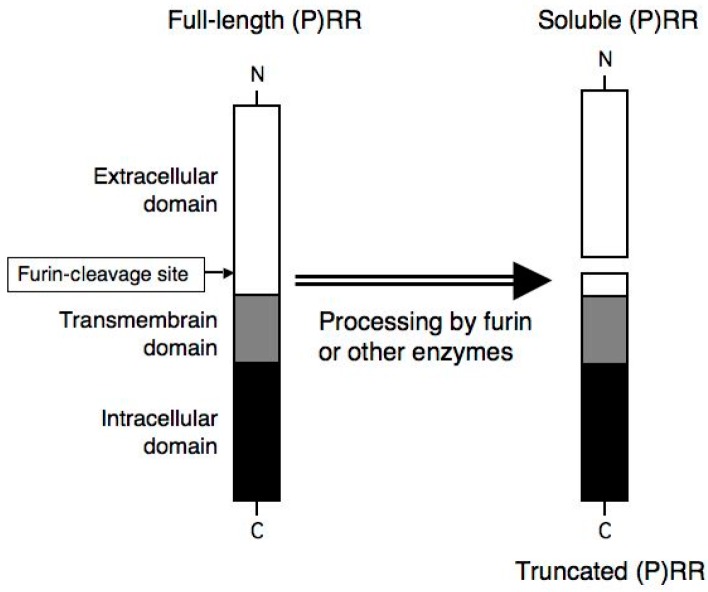
Schematic representation of three molecular forms of (P)RR. Full-length (P)RR consists of extracellular, transmembrane, and intracellular domains. C and N indicate C-terminal and N-terminal of (P)RR protein, respectively. Soluble (P)RR (s(P)RR) and truncated (P)RR are generated from full-length (P)RR by furin cleavage. Other possible processing enzymes to generate s(P)RR include ADAM19 and site-1 protease.

(Pro)renin receptor ((P)RR), a receptor for renin and prorenin, was discovered as a novel component of the renin-angiotensin system (RAS) by Nguyen et al. [1]. (P)RR is a 350-amino-acid protein with a single transmembrane domain (Figure 1) [1,2,3]. The enzymatic activity of prorenin in converting angiotensinogen to angiotensin I is activated non-proteolytically, when prorenin binds to (P)RR [4]. In addition, the binding of renin and prorenin to (P)RR activates (P)RR-mediated intracellular signaling, including extracellular signal-regulated kinase (ERK) 1/2 and Akt [5,6,7]. (P)RR is expressed in various types of cells, such as neurons, endocrine cells, cardiomyocytes, vascular endothelial and smooth muscle cells, and renal tubular cells [3,8,9,10,11,12,13].

Soluble (P)RR (s(P)RR) and a truncated (P)RR are generated from full-length (P)RR by furin cleavage [3,14] (Figure 1). Other possible processing enzymes include ADAM19 [15] and site 1 protease [16]. Nakagawa et al. have recently proposed that s(P)RR is generated by sequential processing by site 1 protease and furin [16]. s(P)RR, comprising the extracellular domain of (P)RR, is secreted to the extracellular space by exocytosis. In contrast, the truncated (P)RR forms the functional complex with vacuolar H^+^-ATPase (v-ATPase). V-ATPase is an ATP-dependent multi-subunit proton pump [17], which plays an essential role in maintaining the acidic environment of intracellular components and the extracellular space. Cell-specific knock-down of the *(P)RR* gene results in loss-of-function of v-ATPase, impaired autophagy and cell death in cardiomyocytes [18], and podocytes in the kidney [19,20]. Thus, (P)RR plays an essential role in the v-ATPase function and survival of cells, at least in cardiomyocytes and podocytes. Moreover, the complex of (P)RR and v-ATPase is involved in the Wnt/β-catenin pathway, which plays essential roles in embryonic development, as well as in the pathophysiology of various diseases including cancers [21].

s(P)RR is present in blood and urine [14]. We have recently reported that plasma concentrations of s(P)RR are elevated in patients with obstructive sleep apnea syndrome (OSAS) in parallel with the severity of disease [22,23]. In this review article, we review plasma concentrations of s(P)RR in patients with various diseases including OSAS, and discuss the possible relation between plasma s(P)RR and oxidative stress.

## 2. Plasma Concentrations of s(P)RR in Various Pathological Conditions

Plasma s(P)RR concentrations were elevated in patients with chronic kidney disease (CKD) in parallel with renal dysfunction [24]. Increased plasma s(P)RR concentrations were also correlated with renal dysfunction in patients with heart failure [25]. Hamada et al. [24] and Fukushima et al. [25] speculated that s(P)RR might be involved in the development and progression of renal injury. Moreover, high serum s(P)RR levels were associated with low ankle-brachial index (an indicator of severe atherosclerosis) in maintenance hemodialysis patients, suggesting that serum s(P)RR reflected atherosclerotic conditions [26].

Watanabe et al. [27] showed that an increase in plasma s(P)RR concentrations during early pregnancy predicted systolic/diastolic blood pressure elevation in later pregnancy, and high s(P)RR concentrations at delivery were significantly associated with preeclampsia. Watanabe et al. [27] speculated that (P)RR is involved in the tissue RAS activation and could also activate prorenin in plasma, thereby leading to the activation of the circulating RAS. Such tissue and circulating RAS activation by (P)RR may underline the mechanism of blood pressure elevation in the later stage of pregnancy [27]. Moreover, Watanabe et al. [28] showed that increased plasma s(P)RR concentrations during the first trimester may predict the development of gestational diabetes mellitus during later pregnancy. This may be due to an association between insulin resistance and (P)RR. The same group showed that higher plasma s(P)RR concentrations in cord blood were associated with appropriate intrauterine fetal growth, suggesting a relationship between s(P)RR and Wnt signaling in fetus development [29].

(P)RR was expressed in various types of tumor cells, such as aldosterone-secreting adenomas [30], breast cancers [31] and pancreatic ductal adenocarcinomas [32]. (P)RR may be related to the proliferation of tumor cells via ERK 1/2 signaling and/or the Wnt/β-catenin pathway. Moreover, plasma s(P)RR concentrations were shown to be elevated in patients with pancreatic ductal adenocarcinoma [32].

In contrast, dehydration for three days in rats decreased plasma s(P)RR levels and expression levels of furin in the kidney, whereas it increased expression levels of full-length (P)RR in the kidney [33]. Thus, water deprivation may downregulate s(P)RR generation from full-length (P)RR by furin, and increased intracellular levels of full-length (P)RR may contribute to the upregulation of the renal RAS system.

These reports indicate that plasma s(P)RR levels reflect the local RAS status, kidney function, diabetes mellitus, cardiovascular tissue damage, or the presence of tumors. By contrast, there is no significant correlation between plasma s(P)RR concentrations and plasma concentrations of renin, prorenin, or aldosterone in healthy subjects and in patients with diabetes mellitus, hypertension, primary aldosteronism, or Gitelman syndrome [34]. The physiological function of s(P)RR remains to be clarified. There have been no reports on positive correlations between plasma s(P)RR levels and plasma renin activity.

## 3. Plasma Concentrations of s(P)RR in Obstructive Sleep Apnea Syndrome

Young et al. reported [35] that the estimated prevalence of sleep-disordered breathing, defined as an apnea-hypopnea index (AHI) of 5 or higher, was 9% for women and 24% for men. Thus, obstructive sleep apnea syndrome (OSAS) is a common disease. OSAS are frequently associated with obesity [36], hypertension [37], diabetes mellitus [38], and the enlarged volume of the soft tissue structures surrounding the upper airway [39]. The endocrine system may play a role in the pathophysiology of OSAS, because the altered plasma levels were observed in the RAS [40,41], atrial natriuretic peptide [42], endothelin-1 [43], vascular endothelial growth factor [44], erythropoietin [44,45], and orexin-A [46,47]. Moller et al. [41] reported that plasma aldosterone levels were elevated and plasma renin activity was suppressed in OSAS patients. In contrast, Svatikova et al. [48] showed no significant changes in plasma aldosterone levels in OSAS patients. Because OSAS patients were frequently accompanied by hypertension and diabetes mellitus, we studied plasma concentrations of s(P)RR in OSAS patients [22,23].

**Figure 2:**
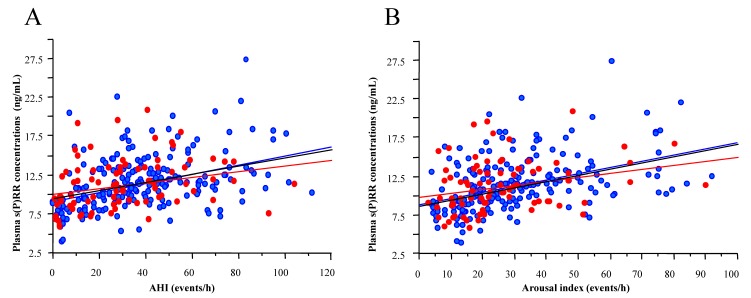
Significant correlations between plasma s(P)RR levels and apnea-hypopnea index (AHI) (**A**); and between plasma s(P)RR levels and arousal index (**B**) in 289 subjects (206 men and 83 women). The subjects comprised of 259 patients with obstructive sleep apnea syndrome (OSAS) and 30 non-OSAS control subjects. The dark lines; trend for all subjects. The blue lines and blue circles; male subjects. The red lines and red circles; female subjects. Reproduced from the Reference [23] with kind permission from the Tohoku University Medical Press, Sendai, Japan.

Plasma concentrations of s(P)RR were studied in 259 OSAS patients (187 men and 72 women) and non-OSAS subjects (19 men and 11 women) [23]. Significant positive correlations were observed between plasma s(P)RR levels and AHI (a marker for the severity of OSAS) (male, *r* = 0.413, *p* < 0.0001; and female, *r* = 0.263, *p* < 0.05) (Figure 2A), and between plasma s(P)RR levels and arousal index (male, *r* = 0.427, *p* < 0.0001; and female, *r* = 0.277, *p* < 0.05) (Figure 2B). Thus, plasma s(P)RR levels were elevated in OSAS patients in parallel with the severity of the disease. The association between s(P)RR levels and the severity of OSAS was higher in male OSAS patients than in female OSAS patients. By contrast, plasma s(P)RR levels were independent of the activity of the circulating RAS; no significant correlation was found between plasma s(P)RR levels and plasma renin activity, or between plasma s(P)RR and aldosterone levels in OSAS patients. These findings are consistent with the report on plasma s(P)RR concentrations by Nguyen et al. [34].

**Figure 3:**
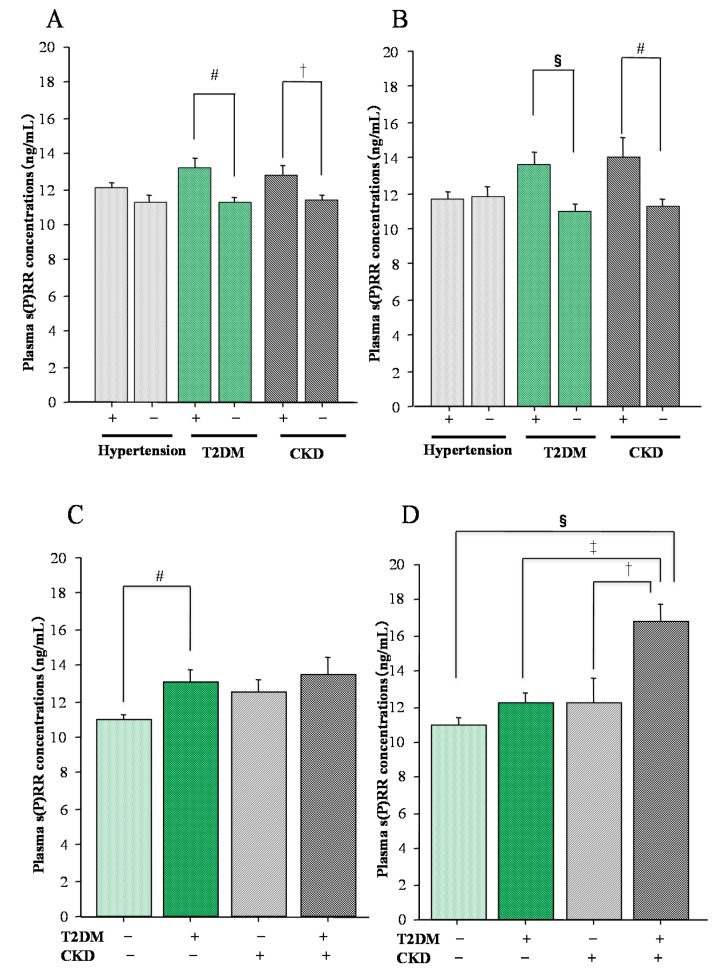
Relation of plasma s(P)RR concentrations with the presence of hypertension, type 2 diabetes mellitus (T2DM), or chronic kidney disease (CKD) in patients with obstructive sleep apnea syndrome (OSAS). (**A**) Male OSAS patients with and without hypertension (*n* = 105 and *n* = 82), with and without T2DM (*n* = 41 and *n* = 146), and with and without CKD (*n* = 39 and *n* = 148); (**B**) Female OSAS patients with and without hypertension (*n* = 35 and *n* = 37), with and without T2DM (*n* = 21 and *n* = 51), and with and without CKD (*n* = 13 and *n* = 59); (**C**) Male OSAS patients without T2DM and CKD (*n* = 119), with T2DM but no CKD (*n* = 29), with CKD but no T2DM (*n* = 27), and with both T2DM and CKD (diabetic kidney disease) (*n* = 12); (**D**) Female OSAS patients without T2DM and CKD (*n* = 44), with T2DM but no CKD (*n* = 15), with CKD but no T2DM (*n* = 8), and with both T2DM and CKD (diabetic kidney disease) (*n* = 5). Data are shown as means ± S.D. († *p* < 0.05; ‡ *p* < 0.01; # *p* < 0.005; § *p* < 0.001). Reproduced from Reference [23] with kind permission from the Tohoku University Medical Press, Sendai, Japan.

The presence of hypertension had no significant effects on plasma s(P)RR concentrations in both male and female OSAS patients (Figure 3A,B). Moreover, the types of anti-hypertensive drugs had no significant effects on plasma s(P)RR concentrations in OSAS patients. On the other hand, the presence of type 2 diabetes mellitus (T2DM) or chronic kidney disease (CKD) affected plasma s(P)RR concentrations in OSAS patients in both sexes (Figure 3A,B). Female OSAS patients with both T2DM and CKD (diabetic kidney disease) showed higher levels of plasma s(P)RR than the other three groups (Figure 3D), whereas male OSAS patients with both T2DM and CKD did not (Figure 3C). These findings suggested that the presence of diabetic kidney disease had a greater effect on plasma s(P)RR concentrations in female OSAS patients.

Continuous positive airway pressure (CPAP) is commonly used for the treatment for moderate or severe OSAS patients. The amelioration of OSAS by CPAP treatment results in decreased plasma s(P)RR concentrations, confirming that plasma s(P)RR concentrations reflect the severity of the OSAS [22,23]. Thus, the most major factor contributing to the elevation of plasma s(P)RR levels in OSAS patients is the severity of the OSAS, which is represented by AHI. Other factors that elevate the levels are CKD and T2DM.

## 4. Oxidative Stress and (P)RR

(P)RR is ubiquitously expressed throughout the body, including the brain, heart, and kidney [3,8,9,10,11,12,13]. Although the source of s(P)RR in plasma has not been determined, s(P)RR may be secreted by various types of cells in the body.

**Figure 4:**
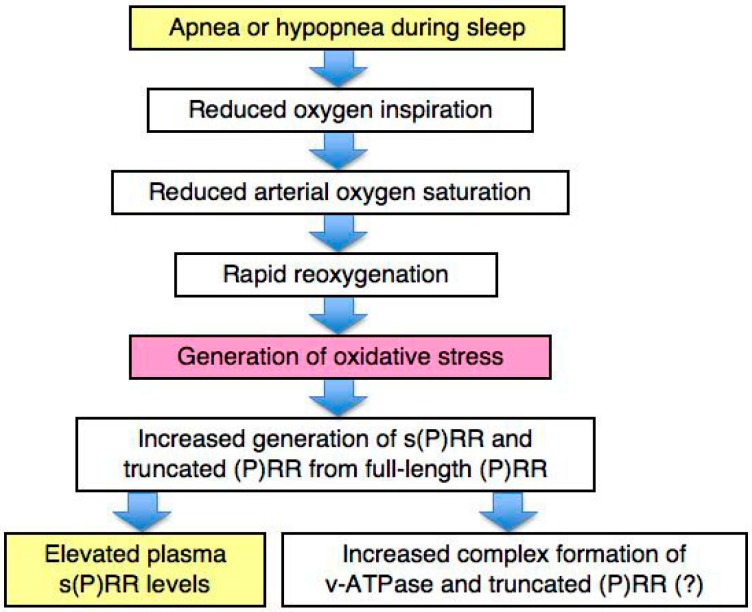
Our hypothesis on s(P)RR and oxidative stress in obstructive sleep apnea syndrome. Apnea or hypopnea during sleep results in reduced oxygen inspiration and reduced arterial oxygen saturation, followed by rapid reoxygenation. Rapid reoxygenation generates oxidative stress, which may increase the generation of s(P)RR and truncated (P)RR from full-length (P)RR. The most important organ for s(P)RR generation by oxidative stress in OSAS may be the brain, followed by the cardiovascular organs. Elevated plasma s(P)RR levels may represent the other aspect of increased complex formation of v-ATPase and truncated (P)RR, which has not been proved yet, however (shown by (?) in the figure).

OSAS is characterized by intermittent hypoxia, which leads to oxidative stress, particularly in the brain and cardiovascular organs [49,50]. The brain may be one of the major organs which are strongly affected by hypoxia and subsequent oxidative stress during sleep apnea and hypopnea. Immunocytochemical studies show that (P)RR is expressed in the neurons of the human hypothalamus [10]. Moreover, Almendros et al. studied tissue oxygenation of the brain, muscle, and fat in a rat model of sleep apnea, and found that arterial oxygen saturation increased quickly in the brain after hypoxemia during obstructive apneas, possibly resulting in increased oxidative stress in the brain [51]. By contrast, such an increase in arterial oxygen saturation after hypoxemia was not found in muscle or fat in a rat model of sleep apnea. We therefore hypothesize that oxidative stress in the brain may elevate plasma s(P)RR concentrations in OSAS patients (Figure 4). We cannot deny the possibility, however, that the generation of s(P)RR is enhanced by hypoxia or oxidative stress in other organs, such as the heart and vascular vessels.

It is noteworthy that v-ATPase activity protects cells from endogenous oxidative stress, and the loss of v-ATPase activity results in chronic oxidative stress [52]. Indeed, treatment with bafilomycin A1, an inhibitor of v-ATPase, increases expression levels of s(P)RR, but not full-length (P)RR in K562 human erythroleukemia cells [53]. To protect against oxidative stress, the brain may require increased activity of v-ATPase, which could therefore promote functional complex formation of v-ATPase and truncated (P)RR. On the other hand, truncated (P)RR forms a functional complex with v-ATPase in this condition, therefore s(P)RR may be secreted increasingly, possibly by various types of cells in the brain, including neurons. Further studies such as cell culture experiments may reveal the mechanism in which hypoxia and subsequent oxidative stress affect the expression of (P)RR and/or the generation of s(P)RR from full-length (P)RR.

## 5. Relationship between (P)RR Mutations and Brain Function

The Ohasama study showed that the polymorphism in the *(P)RR* gene, intervening sequence (IVS)5+169C>T (rs5918007), was associated with ambulatory blood pressure in men [54]. Moreover, the polymorphism of the *(P)RR* gene, +1513A>G (rs6609080), was associated with lacunar infarction and left ventricular hypertrophy in Japanese women [55]. These findings suggest that the polymorphisms in the *(P)RR* gene are related to the pathogenesis of hypertension and its cardiovascular complications.

In contrast, a unique mutation (c.321C>T, p.D107D) in the *(P)RR* (*ATP6AP2*) gene was shown in patients with X-linked mental retardation and epilepsy [56], suggesting the importance of (P)RR in cognitive function. Moreover, another mutation (c.345C>T, p.S115S) in the *(P)RR* gene was shown in patients with X-linked parkinsonism with spasticity [57]. These reports indicate that (P)RR function is essential for brain function, such as cognitive and motor functions. Indeed, (P)RR mRNA is widely expressed in the human brain [10]. In this regard, the functional complex formation of (P)RR and v-ATPase is noteworthy, because v-ATPase plays essential roles in the processing, neurotransmission, and release of neurotransmitters in the nervous system. It remains to be clarified whether oxidative stress caused by impaired v-ATPase activity can explain the pathophysiology of the brain in patients with *(P)RR* mutations. Abnormal control in blood pressure has not been reported in these patients with *(P)RR* gene mutations, suggesting that these mutations do not affect the downstream RAS system.

## 6. Conclusions

Plasma s(P)RR levels are elevated in OSAS patients in parallel with the severity of OSAS. The association of plasma s(P)RR levels with the severity of the condition is higher in male OSAS patients. Oxidative stress in the brain may be increased by oxygenation after apnea in OSAS patients. We hypothesize that oxidative stress, perhaps in the brain, may be one of the candidate factors that elevate plasma s(P)RR levels in OSAS patients. It is tempting to speculate that the brain may require increased activity of v-ATPase via functional complex formation with truncated (P)RR in order to protect against oxidative stress.

## Abbreviations

AHI	Apnea-hypopnea index
CKD	Chronic kidney disease
CPAP	Continuous positive airway pressure
ERK	Extracellular signal-regulated kinase
OSAS	Obstructive sleep apnea syndrome
(P)RR	(Pro)renin receptor
RAS	Renin-angiotensin system
s(P)RR	Soluble (pro)renin receptor
T2DM	Type 2 diabetes mellitus
v-ATPase	Vacuolar H^+^-ATPase
